# Optimizing mRNA-Loaded Lipid Nanoparticles as a Potential Tool for Protein-Replacement Therapy

**DOI:** 10.3390/pharmaceutics16060771

**Published:** 2024-06-06

**Authors:** Rocío Gambaro, Ignacio Rivero Berti, María José Limeres, Cristián Huck-Iriart, Malin Svensson, Silvia Fraude, Leah Pretsch, Shutian Si, Ingo Lieberwirth, Stephan Gehring, Maximiliano Cacicedo, Germán Abel Islan

**Affiliations:** 1 Children’s Hospital, University Medical Center of the Johannes, Gutenberg University, Langenbeckstr. 1, 55131 Mainz, Germany; gambaror@uni-mainz.de (R.G.); ignaciob@uni-mainz.de (I.R.B.); mj.limeres@uni-mainz.de (M.J.L.); malin.svensson@uni-mainz.de (M.S.); fraudesi@uni-mainz.de (S.F.); pretsch@uni-mainz.de (L.P.); stephan.gehring@uni-mainz.de (S.G.); 2 CINDEFI—Centro de Investigación y Desarrollo en Fermentaciones Industriales, Laboratorio de Nanobiomateriales (Universidad Nacional de La Plata (UNLP)-Consejo Nacional de Investigaciones Científicas y Técnicas (CONICET) LA PLATA), Facultad de Ciencias Exactas UNLP, La Plata 1900, Buenos Aires, Argentina; 3 Instituto de Tecnologías Emergentes y Ciencias Aplicadas (ITECA), Universidad Nacional de San Martín (UNSAM)-CONICET, Escuela de Ciencia y Tecnología (ECyT), Laboratorio de Cristalografía Aplicada (LCA), Campus Miguelete, San Martín 1650, Buenos Aires, Argentina; chuck@cells.es; 4 ALBA Synchrotron Light Source, Carrer de la Llum 2–26, Cerdanyola del Vallès, 08290 Barcelona, Spain; 5 Max Planck Institute for Polymer Research, Department of Physical Chemistry of Polymers, Ackermannweg 10, 55128 Mainz, Germany; sis2@mpip-mainz.mpg.de (S.S.); lieberw@mpip-mainz.mpg.de (I.L.)

**Keywords:** lipid nanoparticles, mRNA delivery, cytokines, PBMCs, in vivo biodistribution, metabolic diseases

## Abstract

Lipid nanoparticles (LNPs) tailored for mRNA delivery were optimized to serve as a platform for treating metabolic diseases. Four distinct lipid mixes (LMs) were formulated by modifying various components: LM1 (ALC-0315/DSPC/Cholesterol/ALC-0159), LM2 (ALC-0315/DOPE/Cholesterol/ALC-0159), LM3 (ALC-0315/DSPC/Cholesterol/DMG-PEG2k), and LM4 (DLin-MC3-DMA/DSPC/Cholesterol/ALC-0159). LNPs exhibited stability and homogeneity with a mean size of 75 to 90 nm, confirmed by cryo-TEM and SAXS studies. High mRNA encapsulation (95–100%) was achieved. LNPs effectively delivered *EGFP*-encoding mRNA to HepG2 and DC2.4 cell lines. LNPs induced cytokine secretion from human peripheral blood mononuclear cells (PBMCs), revealing that LM1, LM2, and LM4 induced 1.5- to 4-fold increases in IL-8, TNF-α, and MCP-1 levels, while LM3 showed minimal changes. Reporter mRNA expression was observed in LNP-treated PBMCs. Hemotoxicity studies confirmed formulation biocompatibility with values below 2%. In vivo biodistribution in mice post intramuscular injection showed significant mRNA expression, mainly in the liver. The modification of LNP components influenced reactogenicity, inflammatory response, and mRNA expression, offering a promising platform for selecting less reactogenic carriers suitable for repetitive dosing in metabolic disease treatment.

## 1. Introduction

Over the past three years, the rapid development and global utilization of mRNA vaccines have played a unique role in combating the COVID-19 pandemic. The success of nucleoside-modified mRNA-LNP vaccines, developed by Moderna and Pfizer/BioNTech against severe acute respiratory syndrome coronavirus 2 (SARS-CoV-2), has marked a significant milestone in this field. These licensed vaccines have demonstrated the potential of LNPs as excellent vehicles for delivering nucleoside-modified mRNA [1].

mRNA technology has sparked interest and research into the LNP field as an integral carrier facilitating mRNA delivery into cells. Comprising ionizable lipids, phospholipids, cholesterol, and polyethylene glycol (PEG) lipids, LNPs have been proven effective in encapsulating RNA, ensuring transport to the cytoplasm, and stabilizing its overall structure. The distinct roles of these lipid components underline their significance in enhancing the efficacy and stability of LNPs [2]. 

However, the complex interaction between LNPs and the immune system introduces a dual-edged scenario. Although the intrinsic adjuvant activity of LNPs constitutes an advantage when seeking to formulate vaccines, this activity can interfere with the expression of the protein encoded in the carried mRNA [3]. Additionally, it may constitute a major impediment in the development of mRNA therapies that require repeated administration, such as protein-replacement therapies. Given the intricacies of the immune system, a deeper understanding of how LNPs impact physiology is essential. Moreover, the establishment of techniques for precisely controlling LNP’s effects on the immune system is crucial for advancing the field [4].

Interestingly, LNPs have been used to deliver mRNA constructs encoding enzymes for the treatment of different metabolic diseases [5,6]. The delivered mRNA molecules showed a quick onset of effect, even in only a few hours after their administration. However, in an attempt to reduce the intrinsic reactogenicity of the mRNA, non-immunogenic LNPs are also necessary to prevent mRNA degradation, avoid sensitization, and reduce the side effects of repetitive administrations [7]. In this landscape, the pursuit for optimal formulations becomes a priority, aligning with the broader goal of enhancing therapeutic efficacy while minimizing reactogenicity [8].

The present study explores the intricate interplay between LNP formulations and immune cells, together with the subsequent effects on mRNA translation. A deep study of the complex immunological responses to various LNPs holds the key to refining delivery strategies and unlocking the full potential of precision medicine. Particularly, in vitro cytokine production by PBMCs has emerged as a reliable tool to measure the immune profile induced by LNP-associated effects, offering a window into cellular response dynamics [9]. This research presents novel tailored strategies harnessing the full potential of LNPs in the burgeoning landscape of mRNA therapeutics in metabolic diseases.

## 2. Materials and Methods

### 2.1. Materials 

The (4-hydroxybutyl)azanediyl)bis(hexane-6,1-diyl)bis(2-hexyldecanoate) (ALC-0315), (6Z,9Z,28Z,31Z)-heptatriacont-6,9,28,31-tetraene-19-yl 4-(dimethylamino) butanoate (DLin-MC3-DMA) and alpha-[2-(ditetradecylamino)-2-oxoethyl]-omega-methoxy-poly(oxy-1,2-ethanediyl) (ALC-0159) were purchased from Cayman Chemical (Ann Arbor, MI, USA). 1,2-distearyol-sn-glycero-3-phosphoethanolamine (DSPC), 1,2-Dioleoyl-sn-glycero-3-PE (DOPE), and DMG-PEG 2000, and other lipids, were purchased from Avanti Polar Lipids (Alabaster, AL, USA). CleanCap^®^ Enhanced Green Fluorescent Protein (*EGFP*) mRNA and Firefly Luciferase (*Luc*) mRNA were obtained from TriLink BioTechnologies (San Diego, CA, USA). The GenVoy-ILM™ (GV) lipid mix was purchased from Precision NanoSystems Inc. (Vancouver, BC, Canada). Trypsin (TrpLE™ Express 1X) was obtained from Gibco (ThermoFisher, Waltham, MA, USA). 

### 2.2. Synthesis of LNP Formulations from Different Lipid Mixes (LM)

LNPs were prepared using a self-assembly process by mixing an aqueous solution of the mRNA (*EGFP* or *Luc*) at 120 µg/mL and pH 4.0 (sodium acetate buffer 50 mM), with an ethanolic phase containing the lipids at 12.5 mM using the NanoAssemblr^®^ microfluidic platform (Precision NanoSystems Inc., Vancouver, BC, Canada). The ethanol and aqueous phases were mixed at a total flow rate (TFR) of 12 mL/min with a N/P ratio of 6 and flow rate ratio (FRR) of 3:1 (aqueous: organic). The initial and final waste volumes were 200 µL and 50 µL, respectively. The different LMs were prepared by changing the types of components (ionizable cationic lipid, phosphatidylcholine, cholesterol, and PEG–lipid). The exact composition and molar proportions for each lipid mixture are further discussed in the Section 3.

After synthesis, LNPs were diluted 1:20 (for in vitro) and 1:40 (for in vivo) in 1X phosphate-buffered saline (PBS) and concentrated in Amicon^®^ centrifugal filters 50,000 MWCO (Merck KGaA, Darmstadt, Germany) at 2000× *g* for 5 min per fraction. Finally, the obtained solution was filtered through a 0.22 μm filter and stored at 4 °C for further use. 

### 2.3. Determination of mRNA Encapsulation Efficiency (EE) by Ribogreen Assay 

mRNA was measured by fluorescence intensity with Ribogreen reagent (Thermo-Fischer, Waltham, MA, USA) at 535 nm (emission) with excitation at 485 nm on a TECAN Spark^®^ (Männedorf, Switzerland) plate reader. Calibration curves were generated with the rRNA provided with the Ribogreen kit.

To measure mRNA encapsulation efficiency, the mRNA concentration in the LNP samples was measured in two conditions, with and without incubation in 2% Triton X-100 at 37 °C for 10 min, to measure total mRNA and unencapsulated mRNA, respectively. The difference between the two readings was considered as the encapsulated mRNA.

### 2.4. Particle Size, Zeta Potential (Z-Pot), and Polydispersity Index (PDI)

Measurements were taken in 1/100 PBS dilutions of each formulation. The mean hydrodynamic diameter and size distribution (PDI) of the LNP formulations were determined in triplicates by dynamic light scattering (DLS) in a Nano ZS Zetasizer (Malvern Instruments Corp., Malvern, UK) in ZEN0040 disposable cells. Z-pots for the different LNP formulations were measured with the same equipment in DTS1080 disposable capillary cells. The stability of the formulations was followed up to one month each week by testing changes in size, Z-pot, and EE after storage in a fridge (4 °C).

### 2.5. Cryogenic Transmission Electron Microscopy (Cryo-TEM)

The size, morphology, and distribution of the LNPs were corroborated by cryo-TEM. For cryo-TEM examination, the samples were vitrified using a Vitrobot Mark V (ThermoFisher, Hilsboro, OR, USA) plunging device. Here, 3 µL of the sample dispersion was applied to a Quantifoil or a lacey carbon-coated TEM grid that had been glow-discharged in an oxygen plasma cleaner (Diener Nano^®^, Diener electronic, Ebhausen, Germany) shortly before. After removing excess sample solution with a filter paper, the grid was immediately plunged into liquid ethane. For the subsequent examination, the specimen was transferred to a TEM (FEI Titan Krios G4, Thermo Fisher Scientific, Naarden, The Netherlands), maintaining cryogenic conditions. 

Conventional TEM imaging was performed using an acceleration voltage of 300 kV. Micrographs were acquired with a 4k Direct Electron Detection Camera (Gatan K3, Pleasanton, CA, USA) under low dose conditions. Images were later analyzed by ImageJ^®^ Software (version 1.54).

### 2.6. Small-Angle X-ray Scattering (SAXS)

The SAXS profiles were recorded with an NCD-SWEET beamline (Project ID 2023067620) from the ALBA Synchrotron Light source, Barcelona, Spain. The incoming energy was set at 10 keV with a sample to detector distance of 3.2 m. Liquid samples were placed under low-scattering polymeric capillaries with a 2.2 mm external diameter and 0.1 mm wall thickness. Two-dimensional patterns were recorded in a Pilatus 1M (Dectris, Baden-Daettwil, Switzerland) detector, and one-dimensional patterns were obtained after azimuthal integration with the pyFAI library [10]. Intensity was expressed as a function of the scattering momentum transfer q ((q=4π/λ sin(θ)), which depends on the incoming wavelength (*λ*) and the scattering angle (2*θ*). For each sample, 10 frames of 10 s were taken after discarding possible radiation damage over the sample. Measures were performed at RT (22 °C).

In order to study the structural difference between formulations, a polydisperse multi-shell spherical particle model was employed to take into account the mean electron density difference from the outer shells of each product [11]. The form factor (*P*) of a single particle could be expressed according to the following equation:

$$P(q,R)={\left[\rho_{1}A(R_{1},q)+\;\sum_{i=2}^{N}\;\rho_{i}A(R_{i},q)+\;\right]}^{2}/\;{\left[\rho_{1}V(R_{1})+\;\sum_{i=2}^{N}\;\rho_{i}V(R_{i})+\;\right]}^{2}$$

where A is the amplitude of a homogeneous particle of radius Ri

$$A(R_{i},q)=4\pi \frac{sin(Rq)-Rqcos(Rq)}{q^{3}}$$

*V* is the volume of a sphere ($4\pi /3\;R^{3}$), and $\rho_{i}$ is the electron density of region *i* of the particle. In the current model, we adapted part of the strategy followed by other authors, where they estimated the radial distribution scattering density for neutrons of a multi-shell particle [12]. In the current study, we considered a bilayer nanoparticle with a homogeneous core; these particles are represented by an average radius (*R_av_*) around a certain standard deviation (*σ*) fixed at 20% using a Gaussian distribution function (*D*). For the bilipidic outer shell, three contributions were considered: two for the high density due to the polar region, and one of low electron density. The thickness of each shell was fixed at 2 nm based on the nature of the lipids and the components. The total scattering intensity is expressed in the following equation:

$$I\;(q)=(cte_{1}+cte_{2}S_{eff}(q))\;\int_{0}^{\infty }\;D(R,R_{av},\;\sigma )\;P(q,R,\;\vec{\rho })\;dR+back$$

where *S_eff_* is the structure factor of a lamellar system (multilamellar). A para-crystal structure factor was used [13]. The disorder parameter for the repeat distance was fixed at 0.01. Under these constraints, the variables were the mean core radius, *R_av_*, the electron density of concentric shells, and the average number of layers.

### 2.7. Evaluation of In Vitro Expression Levels 

The expression levels of a reporter mRNA (*EGFP*) encapsulated into the different LNP formulations were evaluated. Human hepatocellular carcinoma cell line (HepG2) and mouse dendritic cell line (DC2.4) cells were used as models to assess transfection. 

HepG2 cells were cultured in RPMI medium supplemented with 10% heat-inactivated fetal bovine serum (iFBS) and 1% antibiotics (100 U/mL penicillin and 100 μg/mL streptomycin). The cells were incubated at 37 °C in a humidified atmosphere with 5% CO_2_. They were seeded in 24-well culture plates at a density of 2.5 × 10^5^ cells/well and allowed to grow for 24 h. LNP transfection was performed at a dose of 500 ng mRNA/well (1 µg/mL) for 24 h. After incubation, the presence of green fluorescent cells was assessed under an inverted fluorescent microscope (Olympus CKX41, Tokyo, Japan). The supernatant as well as the cells (previously washed with PBS and treated with trypsin) were collected and washed with 2 mL fluorescence-activated cell sorting (FACS) buffer (1X PBS with 2% iFBS) to inactivate trypsin. After centrifugation at 400× *g*, 4 °C for 10 min, the cells were resuspended in 70 µL of FACS buffer and stained with 3 µL of 7-Amino-Actinomycin D (7-AAD) to check viability. Samples were measured using a flow cytometer (LSR II, BD Biosciences, Bedford, MA, USA) and analyzed with FlowJo^TM^ software version 10.8 (Ashland, OR, USA; Becton, Dickinson and Company). 

DC2.4 cells were cultures in RPMI-1640, 10% iFBS, 100 U/mL penicillin, and 100 µg/mL streptomycin (1%), 1% L-glutamine (2 mM), 1X non-essential amino acids, 1 mM HEPES, and 0.0054X β-mercaptoethanol. They were seeded in 24-well culture plates at a density of 4 × 10^4^ cells/well and allowed to grow for 24 h. LNPs were used to treat cells at a dose of 500 ng mRNA/well (1 µg/mL) for 24 h. A volume of 10 µL of the supernatant was collected for TNF-ɑ analysis. The same procedure was applied to HepG2 cells in order to determine the transfection efficiency. mRNA premixed with Lipofectamine™ MessengerMAX Reagent (Invitrogen, Carlsbad, CA, USA) according to the protocol provided by the manufacturer served as the transfection control. 

### 2.8. Isolation, Seeding, and LNP Treatment of PBMCs

Buffy coats were obtained from healthy, voluntary donors through the blood bank of the University Medical Center Mainz, following informed consent: 50 mL of blood was transferred to sterile flasks and diluted with 100 mL of Hank’s balanced salt solution (HBSS). PBMCs were isolated through density gradient centrifugation, utilizing Histopaque^®^. Specifically, 35 mL of the diluted blood sample was layered on top of 15 mL Histopaque^®^ and centrifuged at 900× *g*, RT for 20 min, with no break. The PBMC layer was carefully collected and washed with 50 mL of cold HBSS. Subsequently, centrifugation was performed at 400× *g*, 4 °C for 10 min, repeating the washing step twice. Finally, the resulting pellet was resuspended in 50 mL of MACS buffer (PBS, 2 mM ethylenediaminetetraacetic acid (EDTA), and 0.5% bovine serum albumin (BSA) at pH 7.2) for cell counting with trypan blue. 

For LNP treatment, PBMCs were suspended in X-vivo 15 medium (Lonza, Walkersville, MD, USA) supplemented with 100 U/mL penicillin and 100 µg/mL streptomycin, and subsequently seeded in 48-well culture plates at a concentration of 2.5 × 10^5^ cells/well for a 24 h incubation period. Following incubation, cells were exposed to various LNP formulations (either *EGFP* mRNA or *Luc* mRNA), each containing 4 µg of mRNA, along with their respective controls. Apolipoprotein E3 at 1.0 µg/mL was added to enhance LNP transfection based on a previous screening (Figure S1). After a 24 h incubation period, 30 µL of supernatants was collected for cytokine analysis. In order to assess EGFP expression, cells were detached with 50 µL trypsin treatment (37 °C for 10 min) and washed with FACS buffer for further analysis by flow cytometry. For Luc expression, the Promega Luciferase Assay kit (WI, USA) was used, following the manufacturer’s recommendations in a TECAN Spark^®^ (Männedorf, Switzerland), with an automatic injector. 

### 2.9. Determination of Activation Markers in CD11c^+^ Cells from PBMCs 

After the transfection of PBMCs with different LNP formulations, cells were detached with 50 µL of detachment buffer (PBS, 0.5% BSA, 5 mM EDTA, and 4 mg/mL Lidocaine hydrochloride monohydrate) at 37 °C for 30 min and washed twice with FACS buffer. DCs within the PBMC population were identified through staining with anti-CD11c^+^, and the expression levels of the following co-stimulatory molecules were determined: CD40^+^. CD80^+^, CD86^+^, and HLA-DR^+^, via FACS analysis. Briefly, cells were treated with 50 μL Fc receptor blocker (10% Privigen^®^ Immunoglobulin solution) at 4 °C for 15 min and then stained extracellularly at 4 °C for 30 min, protected from light with anti-human fluorescent dye-conjugated monoclonal antibodies: CD11c (eFluor^TM^ 506) purchased from Invitrogen; CD40 (PE-Cy^TM^7), CD80 (PE), CD86 (BD Horizon^TM^ 450), and HLA-DR (APC) were purchased from BD Biosciences. After staining, cells were washed with FACS buffer, followed by incubation with 5 μL of 7-AAD at RT for 5 min in the dark to assess cell viability. Samples were acquired in an LSR II flow cytometer (BD Biosciences), and data were analyzed with FlowJo^TM^ software version 10.8 (Ashland, OR; Becton, Dickinson and Company). The gating strategy is shown in Figure S2. 

### 2.10. Cytokine Profile

Cell culture supernatants were collected and preserved at −20 °C prior to cytokine measurements. TNF-α, CCL2 (MCP-1), CXCL8 (IL-8), IL-1β, IFN-γ, IL-6, CXCL10 (IP-10), and IL-4 were quantified using multiplex Cytometric Bead Assays (CBA) LEGENDplex™ HU Essential Immune Response Panel for PBMCs supernatants and LEGENDplex™ MU Th Cytokine Panel for DC2.4 cell supernatants (BioLegend, San Diego, CA, USA). Assays were performed following the protocol provided by the manufacturer, and data obtained from the acquisition in an LSR II flow cytometer (BD Biosciences) were analyzed using online LEGENDplex^TM^ data analysis software version 8.0 to obtain cytokine concentrations: https://legendplex.qognit.com/workflow/115752 (accessed on 20 December 2023) and https://legendplex.qognit.com/workflow/121702 (accessed on 13 February 2024).

### 2.11. Hemotoxicity Studies

Heparinized venous blood from healthy donors was used after obtaining the corresponding written informed consent (blood bank of the University Medical Center Mainz). The blood was placed in a six-well culture plate in Ham F12 culture medium containing 10% iFBS and exposed to 15 µL of each LNP formulation at 37 °C for 1 or 24 h. After centrifugation of the whole blood–LNP suspension at 2500× *g*, 37 °C for 5 min, the precipitate was discarded. The proportion of lysed red blood cells was quantified by measuring the released hemoglobin at λ = 540 nm. Hemolysis (100%) was determined by exposing erythrocytes to 1.0% Triton X-100, while the negative control was obtained by the incubation of erythrocytes in PBS.

### 2.12. Ethics Statement

All animal procedures were approved by the local authorities (Landesuntersuchungsamt Rhineland-Palatinate). Ethical approval was granted by the Landesuntersuchungsamt LUA, Koblenz, AK G 19-1-080.

### 2.13. In Vivo Biodistribution Assay

C57BL/6-naïve mice were injected intramuscularly (*i.m.*) in both tibialis anterior muscles with 50 μL (25 μL each) *Luc* mRNA-loaded LNPs at a dose of 7 μg mRNA/mouse. After 6 h and 10 min before image acquisition, 150 µL of sterile filtered luciferin substrate (20 g/L) (IVISBrite^TM^ D-luciferin potassium salt) dissolved in PBS was injected intraperitoneally and the relative luciferase activity was evaluated in vivo in isoflurane–oxygen anesthetized mice using an IVIS Spectrum CT (PerkinElmer, Waltham, MA, USA). After imaging, mice were euthanized by cervical dislocation in order to dissect organs (heart, lungs, liver, spleen, kidneys, and inguinal lymph nodes) for ex vivo imaging. Organs were then weighed, and images were analyzed with Living Image^®^ Software version 4.7 (Caliper Life Sciences, Hopkinton, MA, USA).

### 2.14. Statistical Analysis

One-way ANOVA following Fisher’s LSD test was used to compare among groups. *p*-values < 0.05 (*), *p* < 0.01 (**), *p* < 0.001 (***), and *p* < 0.0001 (****) were considered for significant differences. 

## 3. Results and Discussion

### 3.1. Development and Physicochemical Characterization of LNP Formulations

An array of diverse LNP formulations was developed, aiming for repetitive mRNA administration, as required for long-term treatments. The inherent immunological activity of LNPs can be considered as either beneficial, potentially enhancing vaccine responses, or detrimental, particularly in scenarios involving repeated administration such as in metabolic disease treatments [7]. 

**Figure 1:**
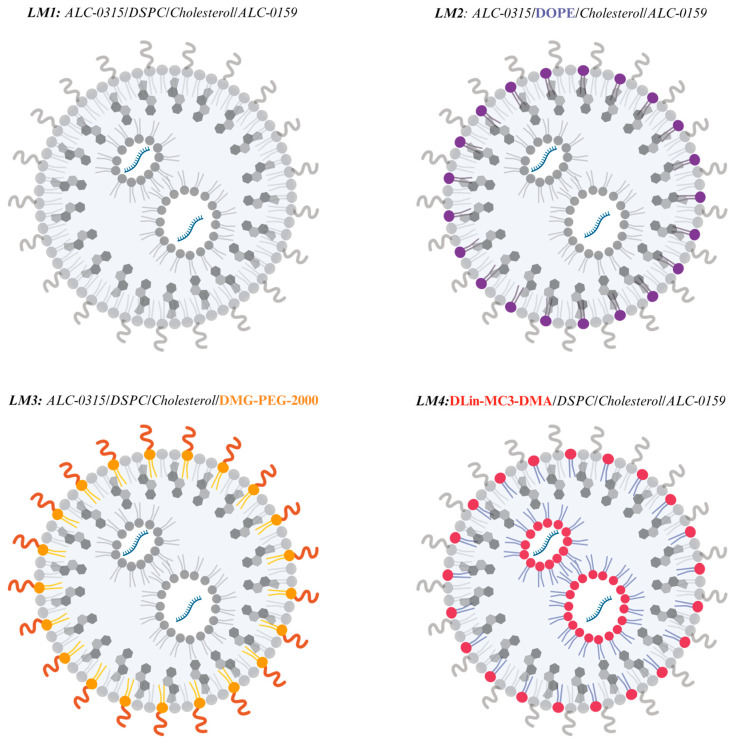
Scheme of the different LNPs from different lipid mixes (LMs) and the modification of each component (created with Biorender). The replacement of each component of LM1 was highlighted with different colors: LM2 (DSPC for DOPE in violet), LM3 (ALC 0159 for DMG-PE-2000 in orange) and LM4 (ALC 0315 for Dlin-MC3-DMA in red).

Initially, the impact of different LNP components on the structure of nanoparticles and the efficacy of mRNA delivery was investigated. LNPs were prepared using a self-assembly process, involving the mixing of an aqueous solution containing the mRNA (*EGFP* or *Luc*) with an ethanolic phase containing lipids, using the microfluidic NanoAssemblr^®^ platform by Precision NanoSystems Inc. The optimization of LNPs was achieved by alternating LNP components and lipid ratios (ionizable lipid, phosphatidylcholine, cholesterol, and PEG–lipid) obtaining four selected and distinct lipid mixes (LMs) (Figure 1).

**Figure 2:**
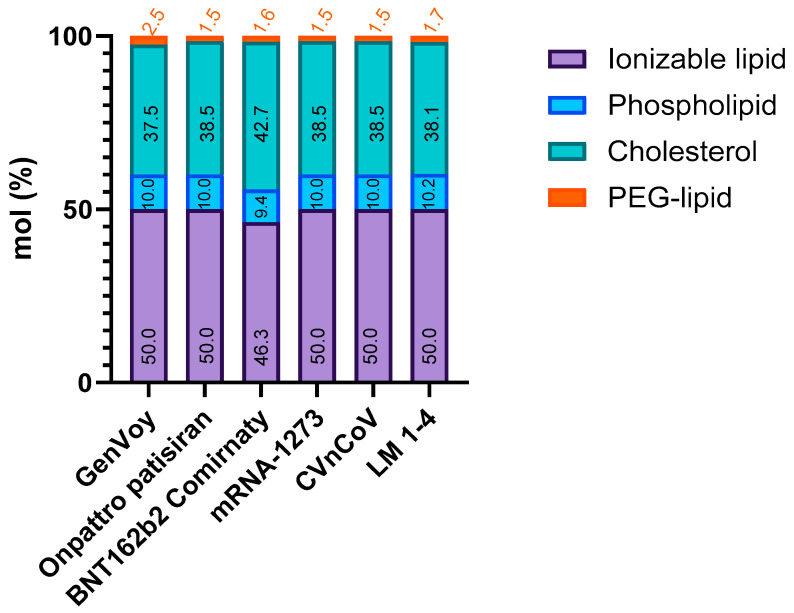
Distribution of the main LNP components by molar fraction: GenVoy, commercial lipid mix provided by Precision NanoSystems, Onpattro (patisiran), BNT162b2 (Comirnaty) Pfizer–BioNTech COVID vaccine, mRNA-1273 Moderna COVID vaccine, CVnCoV Curevac COVID vaccine candidate, and lipid mixes presented in this manuscript (LM 1 to 4).

The main components assessed were selected from lipids available on the market and approved for in vivo experiments. Many of these components were utilized in various licensed mRNA-based vaccines. Figure 2 shows the molar proportions of each type of lipid in some LNP commercial formulations: GenVoy ILM™_research-oriented lipid mix by Precision NanoSystems (now Cytiva); Onpattro (Patisiran), an siRNA formulation from Alnylam (Cambridge, MA, USA) for transthyretin-related hereditary amyloidosis; BNT162b2 (Comirnaty), i.e., Pfizer–BioNTech COVID-19 vaccine; mRNA-1273, i.e., Moderna COVID-19 vaccine; CVnCoV COVID-19 vaccine candidate, and lastly lipid proportions used in this manuscript [14].

**Figure 3:**
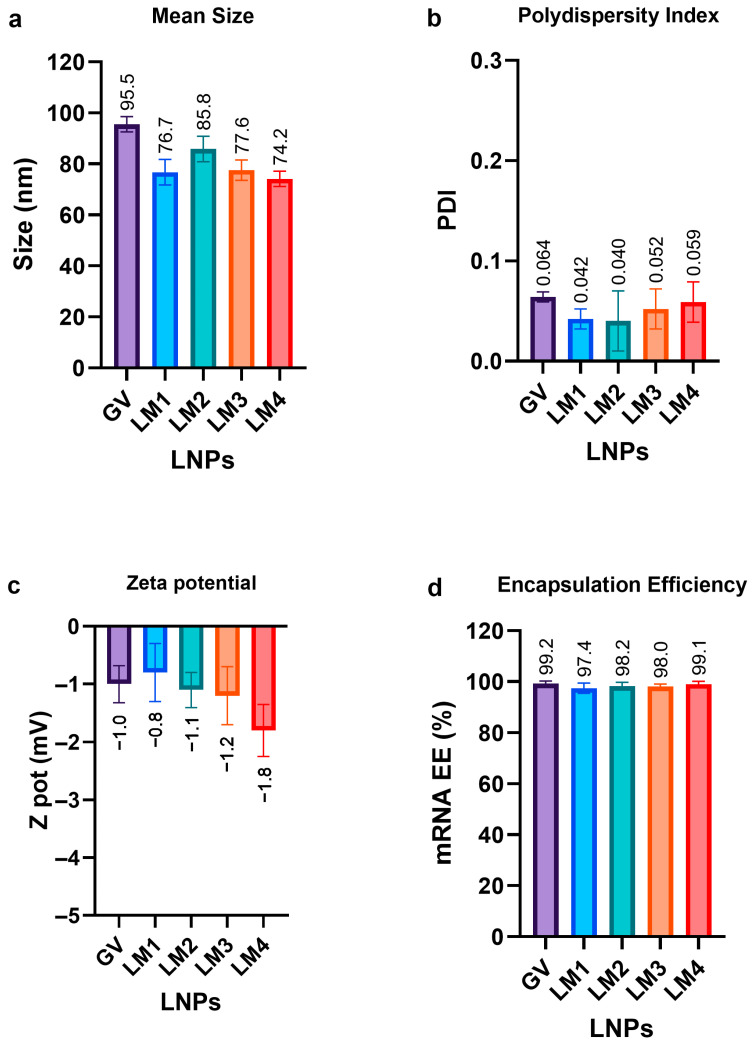
Mean size (**a**), polydispersity index (PDI) (**b**), zeta potential (**c**), and mRNA encapsulation efficiency (EE) (**d**) of the different LNP formulations. Comparison with a commercial formulation (GenVoy) is shown. The graphs represent the mean (n = 3) ± SD.

The proportions selected for our lipid blends represented an estimated average of the proportions used in the commercial formulations. They were similar to those used in some of the products already on the market, with some minor differences [15]. Specifically, the PEG–lipid ratio was kept close to the lower reported values to minimize undesirable side effects associated with dosing [16]. All lipid mixes tested in this study, i.e., GV, LM1, LM2, LM3, and LM4, produced LNPs (i.e., GV-LNP, LM1-LNP, LM2-LNP, LM3-LNP, and LM4-LNP, respectively) of adequate size, PDI, and encapsulation efficiency (Figure 3). 

The mean size of the formulations fell within the range of 75–90 nm, approximately 10 nm smaller than the LNPs produced with the commercial lipid mix (GV). This observation can be explained through the reduced amount (1% less) of PEG–lipid in our LNP formulation, which is crucial for steric stabilization but augments the hydrodynamic ratio [17]. However, PEG is frequently implicated in adverse reactions, such as the generation of PEG-specific antibodies, increased vaccine reactogenicity, and accelerated clearance of other medications containing PEG [16]. Thus, the molar fraction of the PEG–lipid was maintained at approximately 1.7%, aligning with the range observed in other commercially reported formulations, as depicted in Figure 2.

Highly homogeneous LNPs were obtained in all cases, with PDI values lower than 0.1. Zeta potential values of the formulations ranged between −1 and −2 mV, indicating that the LNPs were close to neutrality, as expected at pH 7.4. A high mRNA encapsulation percentage (95–100%) was observed for all formulations, suggesting that changes in the components of the lipid mixes did not adversely affect the LNPs’ ability to successfully carry the mRNA load.

**Figure 4:**
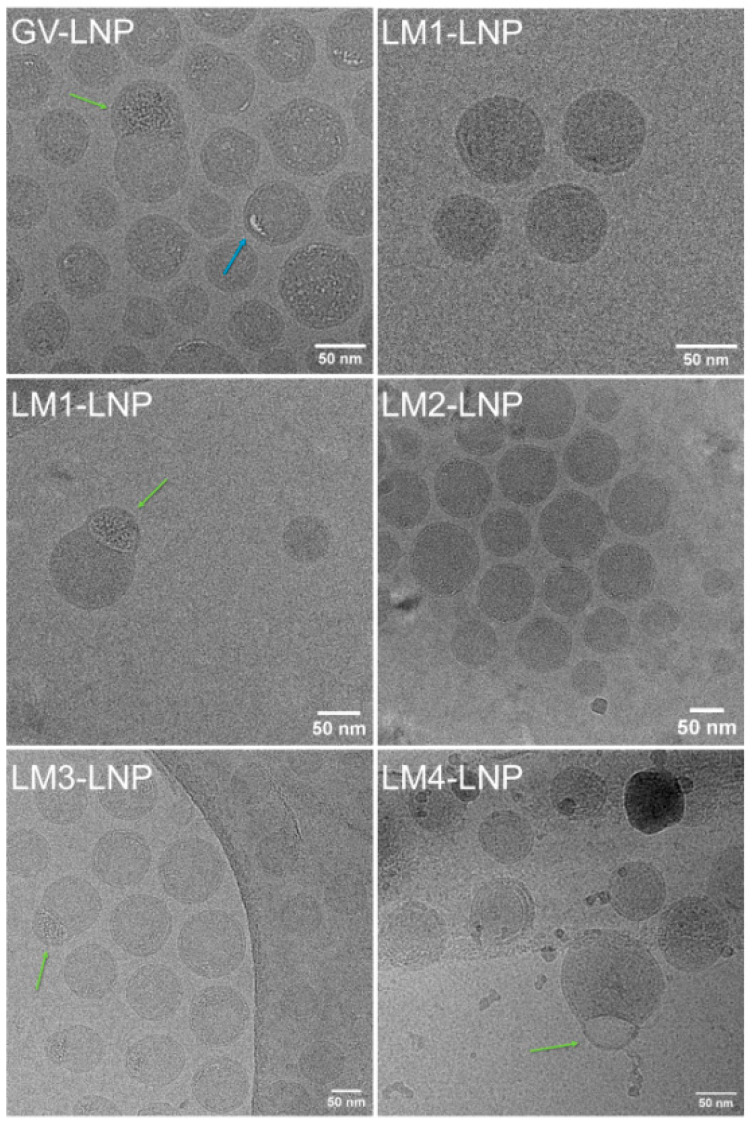
CryoTEM images of the different LNP formulations. Arrows indicate the presence of particular LNP characteristics and irregularities in their structure: “blebs” (green arrows) and internal defects (blue arrows).

The LNP structure was studied through cryo-TEM analysis (Figure 4). The micrographs showed spherical nanoparticles, regular in size and shape. However, irregularities and differences can be found between formulations. GV-LNP showed bilamellar round particles with some internal defects (Figure 4 blue arrow). LM1-LNP showed bilamellar round particles with no internal defects, but like GV-LNP, LM3-LNP and LM4-LNP showed characteristic “blebs” (Figure 4 green arrows), common among formulations with large nucleic acids, such as mRNA. In these LNPs, ionizable lipids are tightly associated with mRNA, and DSPC forms a secondary segregated structure. These “blebs” are mostly absent from LM2-LNP, since DOPE, unlike DSPC, can still form electrodense non-bilayer structures even if excluded from mRNA-ionizable lipid structures [18]. On the other hand, LM4-LNP showed multilamellar particles with inverted micelles structures. This behavior is consistent with small-angle neutron scattering (SANS) analysis, where LNPs containing DLin-MC3-DMA as ionizable lipid showed a core–shell structure [19]. The shell portion of the particle does not contain any mRNA and comprises layers of DSPC, DLin-MC3-DMA, cholesterol, and the hydrophobic portion of the PEG–lipid. 

The formulation obtained with the commercial kit (GV) exhibited nanoparticles in the range of 50–70 nm. Likewise, formulations prepared in our laboratory showed sizes of around 50–70 nm, 50–80 nm, 50–65 nm, and 60–80 nm for LM1-LNP, LM2-LNP, LM3-LNP, and LM4-LNP, respectively. The hydrodynamic diameter (h_D_), as measured by DLS, was consistently higher. This was expected, since h_D_ includes the particle and its solvation sphere. Differences between size as measured by CryoTEM and h_D_ are more evident in the commercial GV-LNP formulation; this can be explained by its comparatively higher PEG–lipid content [20].

**Figure 5:**
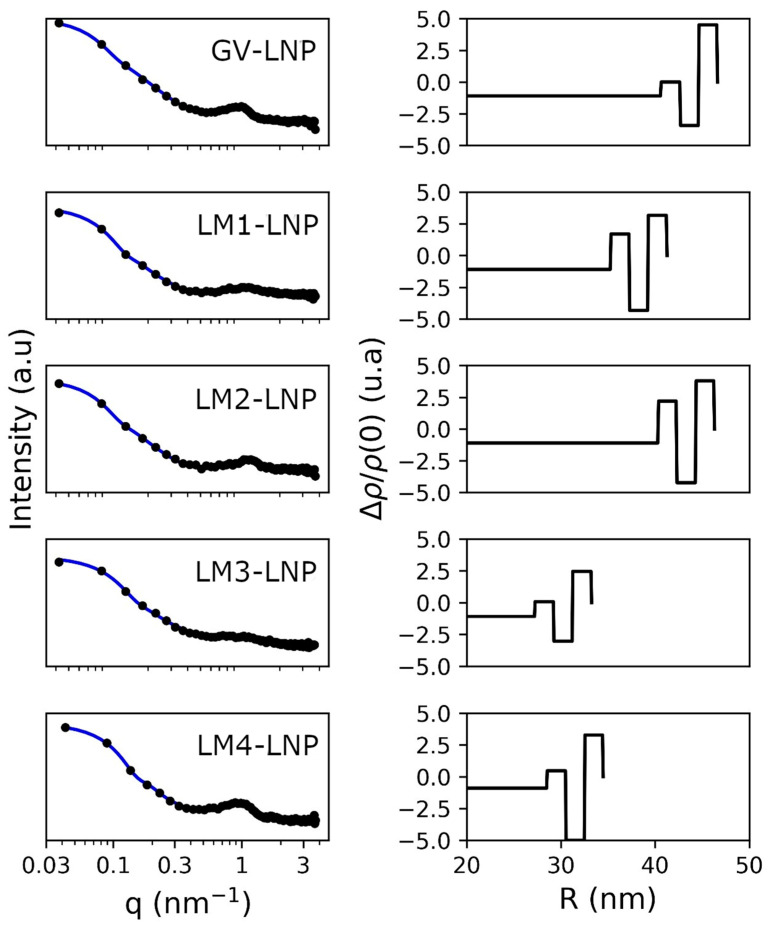
SAXS profiles of the different LNP formulations. The **left** column shows the Log plot of the experimental SAXS pattern in (symbol) and the fitted curve in continuous line. The **right** column shows the electron density profile obtained from the bilayer model.

As a complementary study, SAXS measurements were performed to investigate changes in the crystallographic structure of LNPs (Figure 5).

SAXS patterns were compatible with spherical nanoparticles with a bump near 1 nm^−1^ typically found in bilamellar vesicles or lipidic nanoparticles. There was no evidence of scattering intensity due to the defects observed in a few samples. However, this could be attributed to the low contrast with respect to the rest of the material. The main contribution to the scattering signal was due to size and lipid stacking. The mean size obtained from the small angle region is reported in Table 1. The polydispersity was fixed at 0.2, which was higher than the PDI registered by DLS, but no smearing effects were considered within the simulated intensity, which mainly affects the oscillation due to polydispersity. The bump was more intense for samples GV-LNP, LM2-LNP, and LM4-LNP, which could be attributed to multiple layering with respect to the single bilayer shape (LM3-LNP). In the particular case of LM1-LNP, the structure from the point of view of the SAXS pattern mainly featured a single bilayer particle, although the best result of the fitting was obtained by including this structure factor. The average number of bilayers was near to one, while GV-LNP, LM2-LNP, and LM4-LNP were close to two layers on average (Table 1). 

### 3.2. Stability of LNPs 

**Figure 6:**
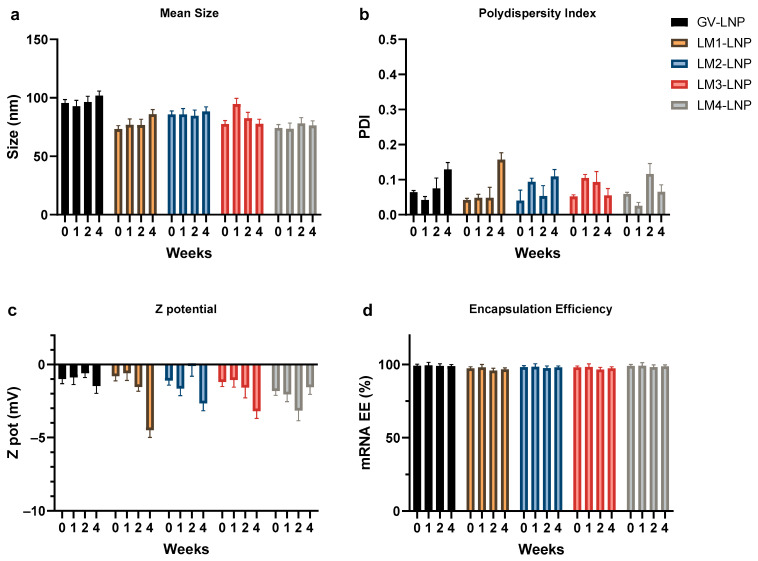
Stability of the formulations after storage at 4 °C for 1 month. The mean size (**a**), polydispersity (PDI) (**b**), zeta potential (**c**), and mRNA encapsulation efficiency (EE) (**d**) were measured every week. The graphs represent the mean (n = 3) ± SD.

The stability of the various formulations was monitored over 1 month following storage at 4 °C (Figure 6). In all instances, the diameter of the LNPs remained stable without significant changes, even after 4 weeks. A minimal increase in the PDI values, ranging from 0.05 to 0.1 units, was observed after only 1 month of storage, suggesting that the presence of aggregates was negligible. Furthermore, all formulations maintained a PDI below 0.15, indicating the high homogeneity of the samples. Concerning the zeta potential, most values remained stable and close to neutrality within the range of −1 to −3 mV. Only LM1 exhibited a notable change, from −1 on day zero to −4 mV after 4 weeks, although this is still within the optimal surface charge range for biological applications. Lastly, no changes in the encapsulation efficiency of mRNA were observed for all tested formulations. This suggests that mRNA remained stable within the LNP structure, protected from degradation by environmental endonucleases.

### 3.3. EGFP mRNA Transfection by LNP Formulations in HepG2 and Dendritic Cell Lines

**Figure 7:**
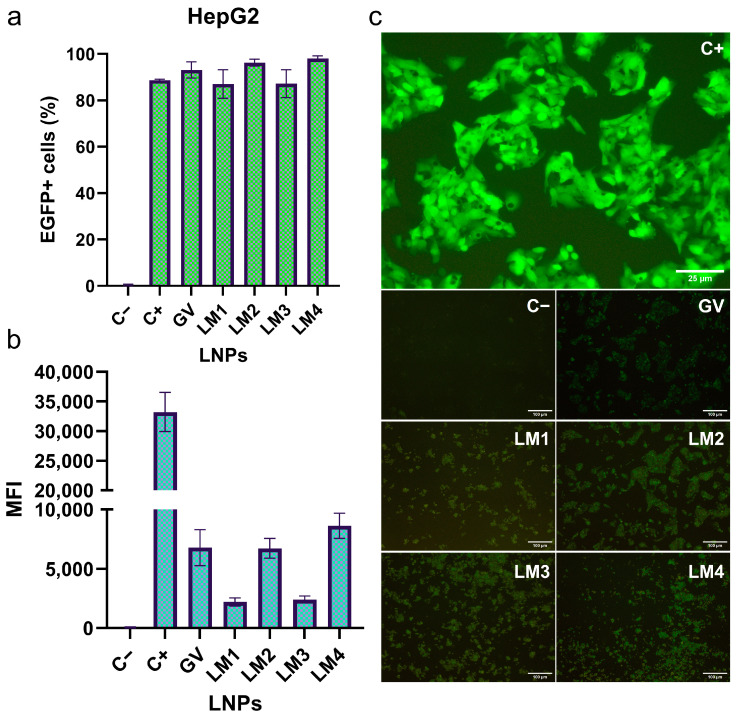
HepG2 cells transfection by the different LM formulations. The percentage of EGFP+ cells (**a**), the mean fluorescence intensity (MFI), (**b**) and fluorescence microscopy images (**c**) were analyzed. The transfection was studied and quantified by the presence of EGFP+ cells (by fluorescence microscopy and flow cytometry). The graphs represent the mean (n = 3) ± SD. Abbreviations: C−: untreated cells; C+: cells treated with *EGFP* mRNA loaded into lipofectamine; GV: commercial formulation; LM: own lipid mixes.

The efficacy of different LNP formulations in transfecting mRNA into eukaryotic cells was assessed (Figure 7). The HepG2 cell line demonstrated a robust transfection efficiency of LNPs. Approximately 90–100% of total cells were successfully transfected using either the commercial formulation or LM1-4 LNPs. While the GV-LNP achieved a transfection rate of 93%, the other formulations exhibited values of 87%, 96%, 87%, and 98% for LM1-LNP to LM4-LNP, respectively. The positive control, the transfection of *EGFP* mRNA using lipofectamine, displayed an 89% transfection rate. It exhibited a stronger green fluorescence signal, however, when compared with other samples, evidenced through fluorescence imaging and the mean EGFP fluorescence intensity measured by flow cytometry. According to flow cytometry results, both GV-LNP and LM2-LNP demonstrated EGFP expression levels approximately 5 times lower than the positive control. LM1-LNP and LM3-LNP displayed even lower expression levels, around 15 times lower than the positive control, while LM4-LNP showed a 4-fold reduction in expression.

These results primarily suggested that the ionizable lipid DLin-MC3-DMA might possess superior transfection ability compared to ALC-0315 in the HepG2 cell line model. Additionally, the replacement of DSPC with DOPE hints at an increase in the expression of the reporter mRNA. 

**Figure 8:**
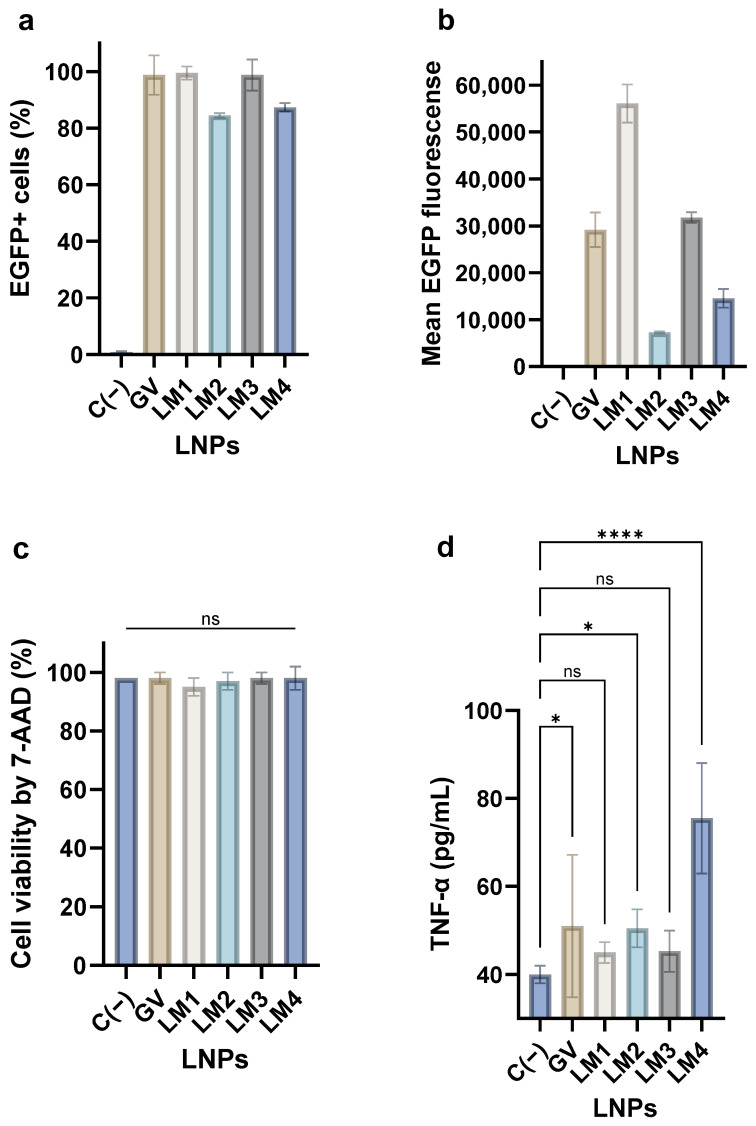
DC 2.4 mouse dendritic cell line transfection by the different LM formulations. The EGFP+ cells (**a**), the mean EGFP fluorescence (**b**), and the cell viability by 7-AAD (**c**) were determined by flow cytometry. The release of TNF-α by LNP-stimulated DC was measured by CBA (**d**). One-way ANOVA following Fisher’s LSD test was used to compare among groups. ns: not significant, *p* < 0.05 (*) and *p* < 0.0001 (****). MFI: mean fluorescence intensity. Abbreviations: C−: untreated cells; GV: commercial formulation; LM: own lipid mixes.

After demonstrating the LM formulation’s efficacy to transfect eukaryotic cells, transfection on a mouse dendritic cell line (DC2.4) was studied as a model of specialized phagocytic cells while expressing an immunologically relevant phenotype (Figure 8). When compared with the results obtained in HepG2 cells, different transfection rates were found in DC2.4 cells for all tested formulations. The GV-LNP, LM1-LNP, and LM3-LNP transfected almost 100% of the cells, while the LM2-LNP and the LM4-LNP showed transfection efficiencies of around 80%. Regarding the mean fluorescence intensity, the LM1-LNP showed a strong fluorescence signal, around double that of the GV-LNP and LM3-LNP formulations. The other two formulations, LM2-LNP and LM4-LNP, displayed an intensity between 2 and 3 times lower than GV-LNPs. The obtained results showed that replacing DSPC with DOPE reduces the targeting of LNPs to dendritic cells. This finding provides evidence about the relevance of the phospholipid role in LNP-mediated mRNA delivery. Additionally, these results may add another dimension to the organ-targeting specificity previously observed by other researchers with DOPE (liver) and DSPC (spleen) [21]. Notably, the viability of the dendric cells was not affected after LNP treatment. 

The discrepancy in transfection efficacy between HepG2 and DC cell lines may be related to the nature of HepG2 cells, which exhibit a hepatocyte-like phenotype and represent a non-immunogenic model for mRNA expression. Therefore, they do not accurately reflect the modulation between transfection efficacy and innate immune response. However, DCs show an immune response to stimuli such as mRNa transfection, resulting in the triggering of an innate response with cytokine release. 

Another interesting observation emerged from the cytokine release profile of DC following LNPs treatment. Particularly, the TNF-α release showed discernible differences in secretion among the various LNP formulations. Notably, LM1-LNP and LM3-LNP, which exhibited higher transfection efficiencies, showed no significant differences (*p* > 0.05) compared to the negative (untreated) control. Conversely, LM2-LNP and, to a greater extent, LM4-LNP, demonstrated 25% and 88% increases, respectively, in TNF-α release compared to the basal DC control. This trend aligns with the formulations displaying lower transfection efficiencies. This correlation between inflammatory response and mRNA expression was in agreement with observations from other authors [8]. 

At this stage, it was confirmed that the performance of our LM-LNPs was comparable to that of the commercial formulation (GV) in terms of physicochemical parameters (size and PDI, morphology), mRNA encapsulation, and transfection efficiency.

### 3.4. mRNA Transfection and Cytokine Secretion by PBMCs Treated with LNPs

**Figure 9:**
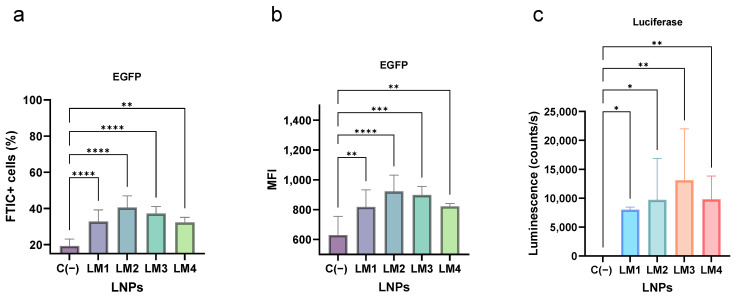
PBMC transfection by the different LM formulations. The mRNA transfection was studied by two techniques: the expression of *EGFP* mRNA detected by flow cytometry (**a**,**b**) and the expression of *Luc* mRNA quantified using a Luciferase kit (**c**). PBMCs were transfected with 8 µg/mL of mRNA and in the presence of ApoE (1 µg/mL). The graphs represent the mean (n = 5) ± SD. One-way ANOVA following Fisher’s LSD test was used to compare among groups. *p* < 0.05 (*), *p* < 0.01 (**), *p* < 0.001 (***), and *p* < 0.0001 (****). MFI: mean fluorescence intensity. Abbreviations: C−: untreated cells; LM: own lipid mixes.

To study in more detail the cytokine profile associated with LNP treatment, PBMCs were transfected with LNP formulations containing a reporter mRNA (either *EGFP* or *Luc*) at a dose of 8 µg/mL of mRNA and in the presence of ApoE (Figure 9). It has been reported that the biodistribution and cellular uptake of LNPs may be influenced by their surface binding to ApoE, which naturally occurs following in vivo administration and leads to a redistribution of lipids within the shell and core of the LNPs [12]. 

In PBMCs, a high concentration of mRNA was used for two reasons. First, PBMCs are difficult to transfect and require a higher concentration of mRNA, in addition to other factors such as ApoE, which are necessary to enhance transfection (see Figure S1). Secondly, the high mRNA concentration is also related to the high lipid concentration (N/P = 6), which is a suitable approach to test the innate immune response of PBMCs with different mixtures of LNPs. This model allows us to establish an interesting relationship between mRNA expression and innate immune response after transfection with the different formulations. 

In the case of *EGFP* mRNA transfection, approximately 30% to 40% of cells exhibited a positive signal, suggesting the successful transfection of PBMCs. However, a 20% occurrence of positive events was observed in the negative control, indicating the presence of an autofluorescence background produced by the PBMCs. In all instances, the mean fluorescence intensity (MFI) of the LNPs showed statistical significance (*p* < 0.05) compared to the negative control. Additionally, the same LNP formulation loaded with *Luc* mRNA yielded nanoparticles with similar characteristics to those loaded with *EGFP* mRNA. Transfection efficiency was assessed in PBMCs, revealing that LNPs effectively delivered and expressed Luciferase. Overall, it was noted that LM3-LNP consistently exhibited a tendency of higher expression levels of the cargo mRNAs compared to other formulations.

**Figure 10:**
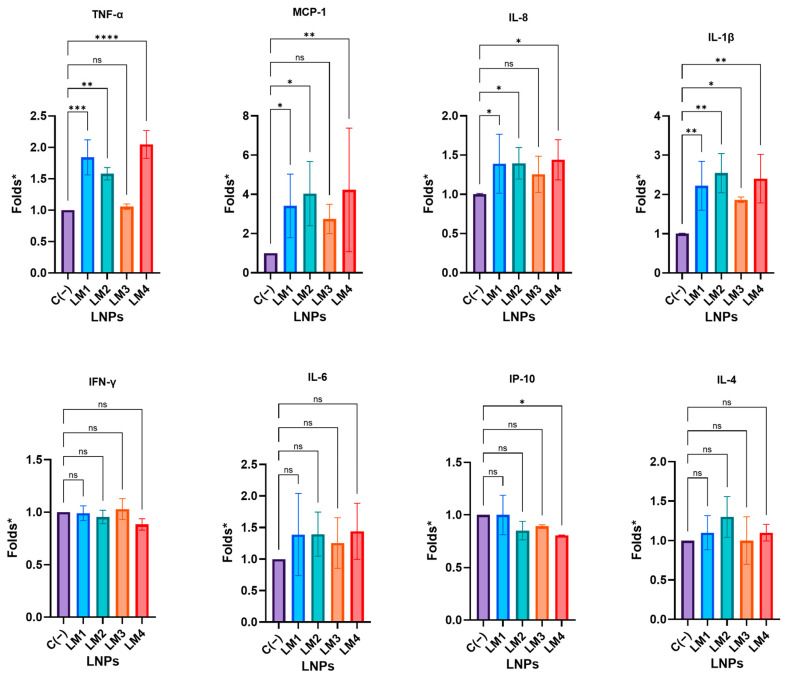
Cytokine secretion of TNF-α, MCP-1, IL-8, IL-1β, IFN-γ, IL-6, IP-10, and IL-4 from LNP-stimulated PBMCs determined by cytometric bead assay. * The results are expressed as folds against the negative control. PBMCs were exposed to LNPs with an equivalent dose of 8 µg/mL of mRNA for 24 h. The graphs represent the mean (n = 5) ± SD. One-way ANOVA following Fisher’s LSD test was used to compare among groups. ns: not significant, *p* < 0.05 (*), *p* < 0.01 (**), *p* < 0.001 (***), and *p* < 0.0001 (****). Abbreviations: C−: untreated cells; LM: own lipid mixes.

Complementarily, the cytokine secretion following exposure to LNPs was also assessed in PBMC supernatants (Figure 10). 

PBMCs have been extensively used as a model to test the inflammatory effects of different molecules [22]. After a 24 h treatment, the levels of TNF-α increased by approximately 1.8, 1.1, and 2.1 times compared to untreated PBMCs (negative control) for LM1-LNP, LM2-LNP, and LM4-LNP, respectively. This finding was indicative of a pro-inflammatory effect of these LNPs. Similarly, the same formulations exhibited 3.4-fold, 4.0-fold, and 4.2-fold increases in Monocyte Chemoattractant Protein 1 (MCP-1), a key player in the inflammation process, attracting and enhancing the expression of other inflammatory factors/cells [23]. Concerning IL-8, the LM1-LNP, LM2-LNP, and LM4-LNP induced a 40% increase in cytokine levels, correlating with the attraction and activation of neutrophils in inflammatory regions [24]. Interestingly, LM3-LNP, wherein the PEG–lipid ALC-0159 was replaced by DMG-PEG 2000, did not exhibit significant differences in the cytokine release of TNF-α, MCP-1, and IL-8 compared to untreated PBMCs. The obtained results strongly imply that LM3-LNP may induce lower inflammatory responses in human cells, presenting a significant aspect for potential applications across various pathologies. A prior study conducted by our group demonstrated the promising potential use of mRNA-LNPs therapy in treating hereditary tyrosinemia 1 or phenylketonuria [5,6]. The imperative requirement for carriers that do not elicit immune or inflammatory responses becomes particularly relevant in long-term treatments, underscoring the suitability of LM3-LNP as a highly promising candidate for such applications.

However, an increase of approximately 2 to 2.5 times in IL-1β was observed for all formulations. This cytokine is a potent pro-inflammatory molecule crucial for host-defense responses to infection and injuries [25]. Furthermore, the impact of LNPs on the release of IFN-γ, IL-6, IP-10, and IL-4 was investigated, but no significant or appreciable changes were observed (*p* > 0.05). 

**Figure 11:**
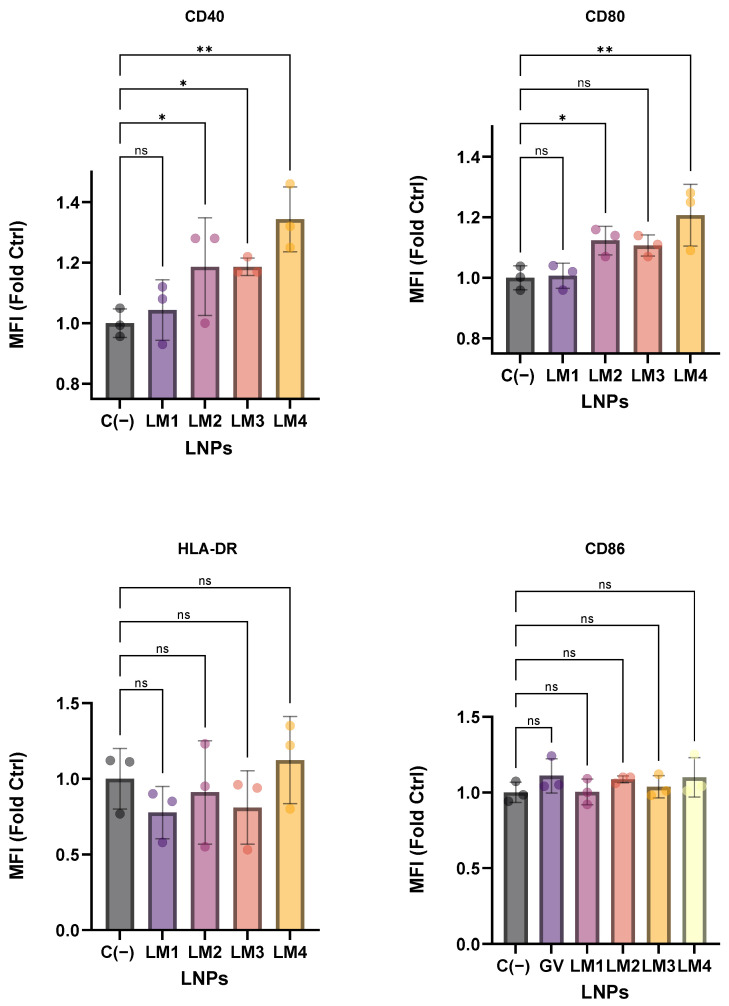
Activation markers of the CD11c+ population from PBMCs after LNP stimulation with the different formulations, determined by flow cytometry analysis. The graphs represent the mean (n = 3) ± SD. One-way ANOVA following Fisher’s LSD test was used to compare among groups. ns: not significant, *p* < 0.05 (*) and *p* < 0.01 (**). Abbreviations: C−: untreated cells; LM: own lipid mixes; MFI: mean fluorescent intensity, folds vs. control (no treated).

Finally, the upregulation of activation markers in the CD11c^+^ population of the PBMCs was studied after treatment with LNPs (Figure 11). 

While no significant differences in the upregulation of HLA-DR and CD86 markers were observed among all tested LNPs (*p* > 0.05), increases in CD40 and CD80 markers were evident for LM2-LNP and LM4-LNP in comparison with the negative control (untreated cells). Specifically, LM4-LNP demonstrated a 30% increase in CD40 expression and a 20% increase in CD80 expression, consistent with its ability to induce higher cytokine secretion. Conversely, LM1-LNP and LM3-LNP resulted in minimal or negligible activation effects in the context of CD40 and CD80 markers. 

### 3.5. Hemotoxicity of the LNPs

Before the in vivo experiment, the biocompatibility of the LNP formulations was assessed by determining hemotoxicity (Table 2). This was the first approach to identifying the potential negative effects of the formulations. The interaction with erythrocytes becomes an important factor when assessing the safety of LNPs [26]. According to the ISO/TR 7406 standard, biomaterials that have less than 5% hemolysis could be safe for biomedical applications [27]. 

After 1 h of exposure to LNPs, no hemotoxicity was observed. Even after 24 h, the degree of hemolysis remained below 2%, indicating that all developed formulations were safe for in vivo application.

### 3.6. In Vivo Biodistribution

**Figure 12:**
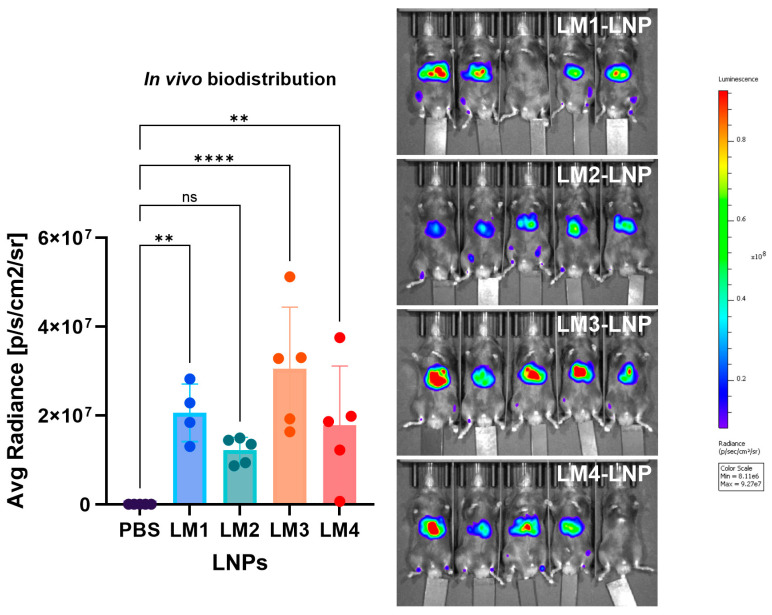
The in vivo biodistribution of the LNPs determined in a C57BL/6-naïve mice model after i.m. injection with the LNP formulations delivering *Luc* mRNA. The graphs represent the region of interest (ROI) mean n = 5 ± SD, except for LM1-LNP, in which n = 4. One-way ANOVA following Fisher’s LSD test was used to compare among groups. ns: not significant, *p* < 0.01 (**), *p* < 0.0001 (****). Abbreviations: PBS: phosphate-buffered-saline-treated mice; LM: mice treated with our own LNP–lipid mixes.

The in vivo biodistribution of LNPs was determined in C57BL/6-naïve mice following *i.m.* injection with *Luc* mRNA-LNPs. Initially, bioluminescence detected for the full body was quantified (Figure 12). In comparison with PBS treatment, all LNP formulations exhibited Luc expression. Although statistical analysis indicated no significant differences for LM2-LNP vs. PBS (*p* > 0.05), clear bioluminescence was observed, with an average radiance in the order of 10^7^. Significant bioluminescence was observed for LM1-LNP and LM4-LNP (*p* < 0.05), while maximum expression was observed with LM3-LNP (*p* < 0.0001). The in vivo results collected for LM3-LNPs were very well correlated. Consistent with the previous in vitro observations, LM3-LNP exhibited low cytokine release, minimal cellular activation, and high mRNA expression levels.

**Figure 13:**
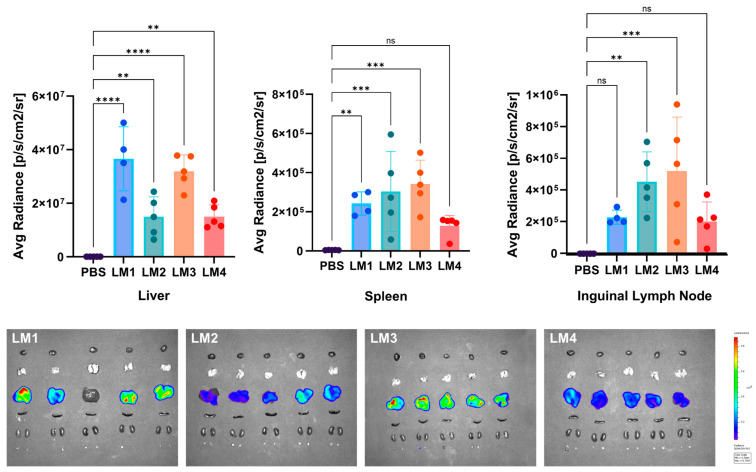
The in vivo biodistribution of the LNPs at organ level in a C57BL/6-naïve mice model after i.m. injection with the LNP formulations delivering *Luc* mRNA. The graphs represent the mean with n = 5 ± SD, except for LM1, in which n = 4. One-way ANOVA following Fisher’s LSD test was used to compare among groups. ns: not significant, *p* < 0.01 (**), *p* < 0.001 (***), and *p* < 0.0001 (****).

On the other hand, the ex vivo imaging was also studied (Figure 13). Consistent with the in vivo observations, ex vivo imaging of the different tissues showed that most of the signal was detected in the liver. LM1-LNP and LM3-LNP exhibited the highest Luc expression in the liver (*p* < 0.0001), while the Luc expression for LM2-LNP and LM4-LNP was approximately three times lower. A significant signal was also found in the spleen, particularly with LM2-LNP and LM3-LNP. These findings align with a study by Pateev et al., which demonstrated that following the administration of LNPs carrying *Luc* mRNA, bioluminescence was predominantly detected in the liver region, with some signal observed in the spleen. This observation can be attributed to the adsorption of four-component LNPs, to ApoE in the bloodstream, leading to their uptake primarily by hepatocytes expressing high levels of low-density lipoprotein receptors [28]. Finally, Luc expression was also observed in inguinal lymph nodes of mice treated with LM2-LNP and LM3-LNP. 

The mRNA for mouse administration was around 7 µg, which was higher than other reports (4 µg/mice [8]; 3 µg/mice [29]), but well under other doses tested for metabolic diseases: 20 µg/mice for hereditary tyrosinemia 1 [5], 60 μg/mice for phenylketonuria [6], or the 10 µg/mice doses used in LNP inflammation studies [30]. Similarly to the approach with PBMCs, high doses of mRNA may be related to high doses of lipids, which represents a suitable situation to study the effect of the lipids on the immune response.

The present results demonstrated that modifications in the composition of LNP formulations result in alterations in biodistribution and mRNA expression across different organs. On the other hand, it was observed that the final biodistribution of the mRNA is not only determined by one lipid component, but also by the overall structure of the LNPs and a combination with other lipids. This might be due to the fact that other influences, such as inflammation, immune stimulation, or lipid interactions, also play a crucial role in mRNA expression and biodistribution. For instance, the inclusion of an ionizable lipid with more immunogenic properties resulted in low expression levels in the liver. This observation was made during the comparison between LM1-LNP (ALC-0315) and LM4-LNP (DLin-MC3-DMA), where the substitution of ALC-0315 with Dlin, an immunogenic ionizable lipid, led to reduced mRNA expression. This heightened reactogenicity was consistent with our measurements of cytokine secretion as well [29]. In another scenario, the replacement of the helper DSPC lipid with DOPE (in LM1 and LM2, respectively) may lead to differences in expression and biodistribution after intramuscular injection. DSPC formulations predominantly express mRNA in the liver, while the DOPE formulation showed a significant signal in the spleen and inguinal lymph nodes. This contrasts with the observations presented by Chandre et al. from intravenous administration, which suggested that DSPC-containing LNPs mostly accumulated in the spleen, whereas identical LNPs substituting DSPC with the helper lipid DOPE preferentially accumulated in the liver [31]. One of the most interesting findings of our study lies in the replacement of the PEG–lipid. Specifically, LM1 contained the PEG–lipid ALC-0159, while LM3 was composed of DMG-PEG2k. Those molecules, with their distinct molecular structures, consist of a lipid structure O-pegylated to a PEG chain with a mass of approximately 2 kDa. The lipid component of ALC-0159 is the N,N-dimyristylamide of 2-hydroxyacetic acid, an amide derivative of 2-hydroxyacetic acid (also known as glycolic acid), combined with two myristyl (tetradecyl) chains. In contrast, DMG-PEG2k comprises myristoyl diglyceride, featuring a glycerol backbone with two fatty acid chains, one of which is myristoyl (tetradecanoyl). This difference in the PEG–lipid nature alone significantly modified the LNP structure, immune response profile, biodistribution, and mRNA expression levels, despite both lipids possessing the same degree of PEGylation and the particles having the same size, which are well-known factors affecting LNP distribution and immunogenicity [4,32].

Considering that most treatments for metabolic diseases involve replacing defective genes with functional enzymes, we have demonstrated that our LNPs successfully express Luc from *Luc* mRNA. This implies the expression of a functional enzyme, a fact that could be extrapolated to certain metabolic diseases, such as phenylketonuria and tyrosinemia, among others [33]. 

As a future perspective, a repetitive injection scheme for LM3-LNPs is being planned for a follow up study, with the concomitant evaluation of LNP-associated inflammation together with mRNA translation efficiency. That scheme was previously developed by our group with a lipidic commercial formulation as a new approach for the treatment of different metabolic diseases [5,6].

## 4. Conclusions

The present study describes a versatile platform for mRNA delivery through the formulation of various LNPs, facilitating controlled effects on different transfected cells. Four distinct LNPs with different compositions were designed, demonstrating high stability, with a mean size ranging from 75 to 90 nm, high homogeneity (PDI < 0.1), and spherical morphology. Alterations in each LNP component resulted in nanoparticles with different crystallographic structures. The zeta potential values indicated near-neutrality particles at pH 7.4, and a remarkable mRNA encapsulation percentage of 95–100%. The efficacy of these LNPs in delivering a reporter mRNA (*EGFP*) to both HepG2 cells and dendritic cells was successfully demonstrated, comparable to the levels of a commercial formulation (GV). Subsequently, the impact of these LNPs on cytokine release in human PBMCs showed that LM1-LNP, LM2-LNP, and LM4-LNP induced significant 1.5- to 4-fold increases in interleukin 8 (IL-8), tumor necrosis factor-alpha (TNF-α), and MCP-1 levels, while LM3-LNP did not elicit significant changes in cytokine release. This suggests that LM3-LNP, composed of ALC-0315/DSPC/Cholesterol/DMG-PEG2k, could be considered less inflammatory. Moreover, LM3-LNP exhibited the highest in vivo expression of *Luc* mRNA after *i.m.* administration, with significant Luc levels detected in the liver, spleen, and inguinal lymph nodes. These findings underscore the potential of modifying various components of LNPs to tailor their immunogenicity and inflammatory response, as well as organ biodistribution. In conclusion, this study offers insights for fine-tuning LNP formulations in the context of mRNA delivery, with potential applications in the treatment of metabolic diseases regarding protein replacement.

## Figures and Tables

**Table 1:** Mean diameter and fitting parameters to the multilayer model of the LNPs determined by SAXS.

Sample	Diameter (nm)	Multilayer (%)	N_av	*χ* ^2^
GV-LNP	84.8 ± 0.1	72	2.3 ± 0.0	0.75
LM1-LNP	82.5 ± 0.2	40	1.2 ± 0.3	0.76
LM2-LNP	97.1 ± 0.2	55	2.2 ± 0.0	0.53
LM3-LNP	67.2 ± 0.2	0	–	0.93
LM4-LNP	57.4 ± 0.1	69	1.7 ± 0.1	0.74

**Table 2:** Hemotoxicity assay of the different LNP formulations.

Hemolysis (%)		
Formulation	Incubation Time (h)	
	1	24
LM1-LNP	0.0 ± 0.0	1.0 ± 0.4
LM2-LNP	0.0 ± 0.0	1.9 ± 0.2
LM3-LNP	0.0 ± 0.0	0.2 ± 0.1
LM4-LNP	0.4 ± 0.6	1.0 ± 0.1

## Data Availability

The original contributions presented in the study are included in the article/supplementary material, further inquiries can be directed to the corresponding author/s (M.C. and G.A.I.).

## Supplementary Materials

The following supporting information can be downloaded at: https://www.mdpi.com/article/10.3390/pharmaceutics16060771/s1, Figure S1: PBMCs transfection; Figure S2: Gating strategy for determination of activation markers of CD11c+ cells from PBMCs.

## References

[1] Reynolds L., Dewey C., Asfour G., Little M. (2023). Vaccine efficacy against SARS-CoV-2 for Pfizer BioNTech, Moderna, and AstraZeneca vaccines: A systematic review. Front. Public Health.

[2] Alameh M.-G., Tombácz I., Bettini E., Lederer K., Ndeupen S., Sittplangkoon C., Wilmore J.R., Gaudette B.T., Soliman O.Y., Pine M. (2021). Lipid nanoparticles enhance the efficacy of mRNA and protein subunit vaccines by inducing robust T follicular helper cell and humoral responses. Immunity.

[3] Li B., Jiang A.Y., Raji I., Atyeo C., Raimondo T.M., Gordon A.G.R., Rhym L.H., Samad T., MacIsaac C., Witten J. (2023). Enhancing the immunogenicity of lipid-nanoparticle mRNA vaccines by adjuvanting the ionizable lipid and the mRNA. Nat. Biomed. Eng..

[4] Lee Y., Jeong M., Park J., Jung H., Lee H. (2023). Immunogenicity of lipid nanoparticles and its impact on the efficacy of mRNA vaccines and therapeutics. Exp. Mol. Med..

[5] Cacicedo M.L., Weinl-Tenbruck C., Frank D., Wirsching S., Straub B.K., Hauke J., Okun J.G., Horscroft N., Hennermann J.B., Zepp F. (2022). mRNA-based therapy proves superior to the standard of care for treating hereditary tyrosinemia 1 in a mouse model. Mol. Ther. Methods Clin. Dev..

[6] Cacicedo M.L., Weinl-Tenbruck C., Frank D., Limeres M.J., Wirsching S., Hilbert K., Pasha Famian M.A., Horscroft N., Hennermann J.B., Zepp F. (2022). Phenylalanine hydroxylase mRNA rescues the phenylketonuria phenotype in mice. Front. Bioeng. Biotechnol..

[7] Córdoba K.M., Jericó D., Sampedro A., Jiang L., Iraburu M.J., Martini P.G.V., Berraondo P., Avila M.A., Fontanellas A., Aranda F., Berraondo P., Galluzzi L. (2022). Chapter Two-Messenger RNA as a personalized therapy: The moment of truth for rare metabolic diseases. International Review of Cell and Molecular Biology.

[8] Zhang H., Han X., Alameh M.-G., Shepherd S.J., Padilla M.S., Xue L., Butowska K., Weissman D., Mitchell M.J. (2022). Rational design of anti-inflammatory lipid nanoparticles for mRNA delivery. J. Biomed. Mater. Res. A.

[9] Friberg D., Bryant J., Shannon W., Whiteside T.L. (1994). In vitro cytokine production by normal human peripheral blood mononuclear cells as a measure of immunocompetence or the state of activation. Clin. Diagn. Lab. Immunol..

[10] Ashiotis G., Deschildre A., Nawaz Z., Wright J.P., Karkoulis D., Picca F.E., Kieffer J. (2015). The fast azimuthal integration Python library: *pyFAI*. J. Appl. Crystallogr..

[11] Pedersen J.S. (1997). Analysis of small-angle scattering data from colloids and polymer solutions: Modeling and least-squares fitting. Adv. Colloid Interface Sci..

[12] Sebastiani F., Yanez Arteta M., Lerche M., Porcar L., Lang C., Bragg R.A., Elmore C.S., Krishnamurthy V.R., Russell R.A., Darwish T. (2021). Apolipoprotein E Binding Drives Structural and Compositional Rearrangement of mRNA-Containing Lipid Nanoparticles. ACS Nano.

[13] Hamley I.W. (2022). Diffuse scattering from lamellar structures. Soft Matter.

[14] Schoenmaker L., Witzigmann D., Kulkarni J.A., Verbeke R., Kersten G., Jiskoot W., Crommelin D.J.A. (2021). mRNA-lipid nanoparticle COVID-19 vaccines: Structure and stability. Int. J. Pharm..

[15] Hald Albertsen C., Kulkarni J.A., Witzigmann D., Lind M., Petersson K., Simonsen J.B. (2022). The role of lipid components in lipid nanoparticles for vaccines and gene therapy. Adv. Drug Deliv. Rev..

[16] Ju Y., Carreño J.M., Simon V., Dawson K., Krammer F., Kent S.J. (2023). Impact of anti-PEG antibodies induced by SARS-CoV-2 mRNA vaccines. Nat. Rev. Immunol..

[17] Ryals R.C., Patel S., Acosta C., McKinney M., Pennesi M.E., Sahay G. (2020). The effects of PEGylation on LNP based mRNA delivery to the eye. PLoS ONE.

[18] Leung A.K.K., Tam Y.Y.C., Chen S., Hafez I.M., Cullis P.R. (2015). Microfluidic Mixing: A General Method for Encapsulating Macromolecules in Lipid Nanoparticle Systems. J. Phys. Chem. B.

[19] Gilbert J., Sebastiani F., Arteta M.Y., Terry A., Fornell A., Russell R., Mahmoudi N., Nylander T. (2024). Evolution of the structure of lipid nanoparticles for nucleic acid delivery: From *in situ* studies of formulation to colloidal stability. J. Colloid Interface Sci..

[20] Maguire C.M., Rösslein M., Wick P., Prina-Mello A. (2018). Characterisation of particles in solution—A perspective on light scattering and comparative technologies. Sci. Technol. Adv. Mater..

[21] Zhang R., El-Mayta R., Murdoch T.J., Warzecha C.C., Billingsley M.M., Shepherd S.J., Gong N., Wang L., Wilson J.M., Lee D. (2021). Helper lipid structure influences protein adsorption and delivery of lipid nanoparticles to spleen and liver. Biomater. Sci..

[22] Eggesbø J.B., Hjermann I., Lund P.K., Joø G.B., Øvstebø R., Kierulf P. (1994). LPS-induced release of IL-1β, IL-6, IL-8, TNF-α and sCD14 in whole blood and PBMC from persons with high or low levels of HDL-lipoprotein. Cytokine.

[23] Singh S., Anshita D., Ravichandiran V. (2021). MCP-1: Function, regulation, and involvement in disease. Int. Immunopharmacol..

[24] Bickel M. (1993). The role of interleukin-8 in inflammation and mechanisms of regulation. J. Periodontol..

[25] Lopez-Castejon G., Brough D. (2011). Understanding the mechanism of IL-1β secretion. Cytokine Growth Factor Rev..

[26] Toledo C., Gambaro R.C., Padula G., Vela M.E., Castro G.R., Chain C.Y., Islan G.A. (2021). Binary Medical Nanofluids by Combination of Polymeric Eudragit Nanoparticles for Vehiculization of Tobramycin and Resveratrol: Antimicrobial, Hemotoxicity and Protein Corona Studies. J. Pharm. Sci..

[27] Chinnaiyan S.K., Karthikeyan D., Gadela V.R. (2018). Development and Characterization of Metformin Loaded Pectin Nanoparticles for T2 Diabetes Mellitus. Pharm. Nanotechnol..

[28] Pateev I., Seregina K., Ivanov R., Reshetnikov V. (2024). Biodistribution of RNA Vaccines and of Their Products: Evidence from Human and Animal Studies. Biomedicines.

[29] Yavuz A., Coiffier C., Garapon C., Gurcan S., Monge C., Exposito J.-Y., Arruda D.C., Verrier B. (2023). DLin-MC3-Containing mRNA Lipid Nanoparticles Induce an Antibody Th2-Biased Immune Response Polarization in a Delivery Route-Dependent Manner in Mice. Pharmaceutics.

[30] Ndeupen S., Qin Z., Jacobsen S., Bouteau A., Estanbouli H., Igyártó B.Z. (2021). The mRNA-LNP platform’s lipid nanoparticle component used in preclinical vaccine studies is highly inflammatory. iScience.

[31] Chander N., Basha G., Yan Cheng M.H., Witzigmann D., Cullis P.R. (2023). Lipid nanoparticle mRNA systems containing high levels of sphingomyelin engender higher protein expression in hepatic and extra-hepatic tissues. Mol. Ther.-Methods Clin. Dev..

[32] Yang M., Zhang Z., Jin P., Jiang K., Xu Y., Pan F., Tian K., Yuan Z., Liu X.E., Fu J. (2024). Effects of PEG antibodies on *in vivo* performance of LNP-mRNA vaccines. Int. J. Pharm..

[33] Marchetti M., Faggiano S., Mozzarelli A. (2022). Enzyme Replacement Therapy for Genetic Disorders Associated with Enzyme Deficiency. Curr. Med. Chem..

