# Supplementary material for: Optimizing mRNA-Loaded Lipid Nanoparticles as a Potential Tool for Protein-Replacement Therapy
Source: Pharmaceutics. 2024 Jun 6;16(6):771. doi: 10.3390/pharmaceutics16060771 (PMC11207542; doi:10.3390/pharmaceutics16060771)
Supplement: Supplementary file 1 [file pharmaceutics-16-00771-s001.zip › pharmaceutics-3036982-supplementary.pdf]

Supplementary material

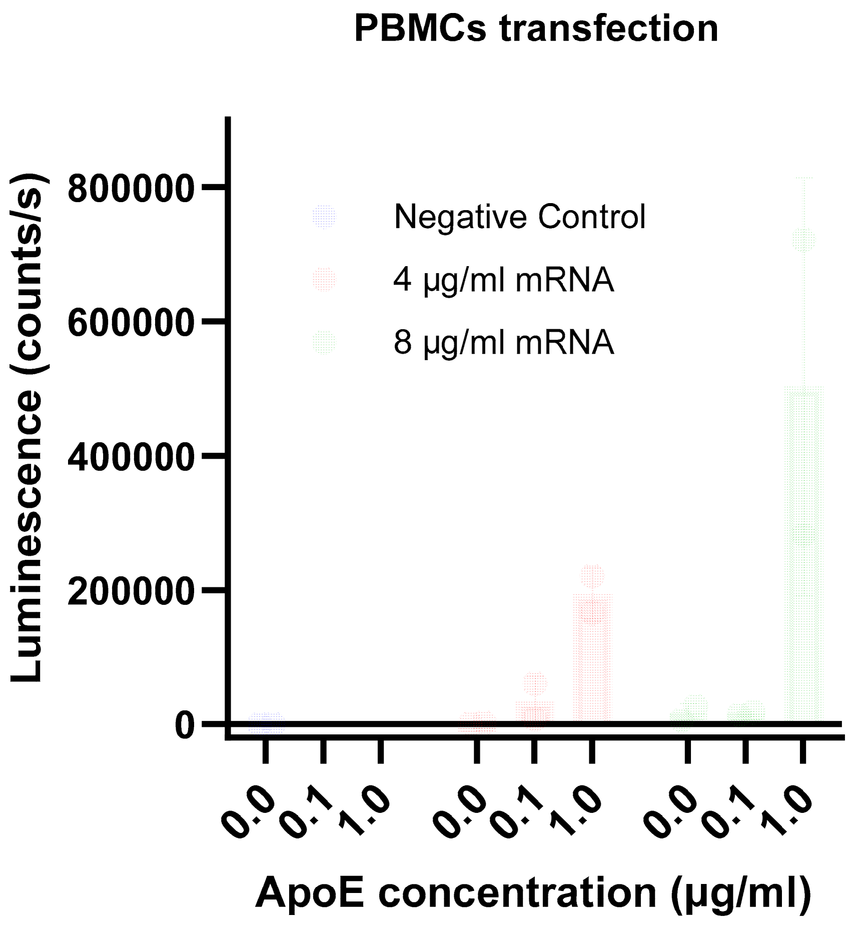

Figure S1 *Luc* mRNA transfection of PBMCs with LNPs (GV formulation) in presence of different Apolipoprotein E3 (ApoE3) concentrations and at two mRNA concentrations (4 and 8 µg/ml) after 24 h incubation. Cell transfection was determined by Luciferase Assay.

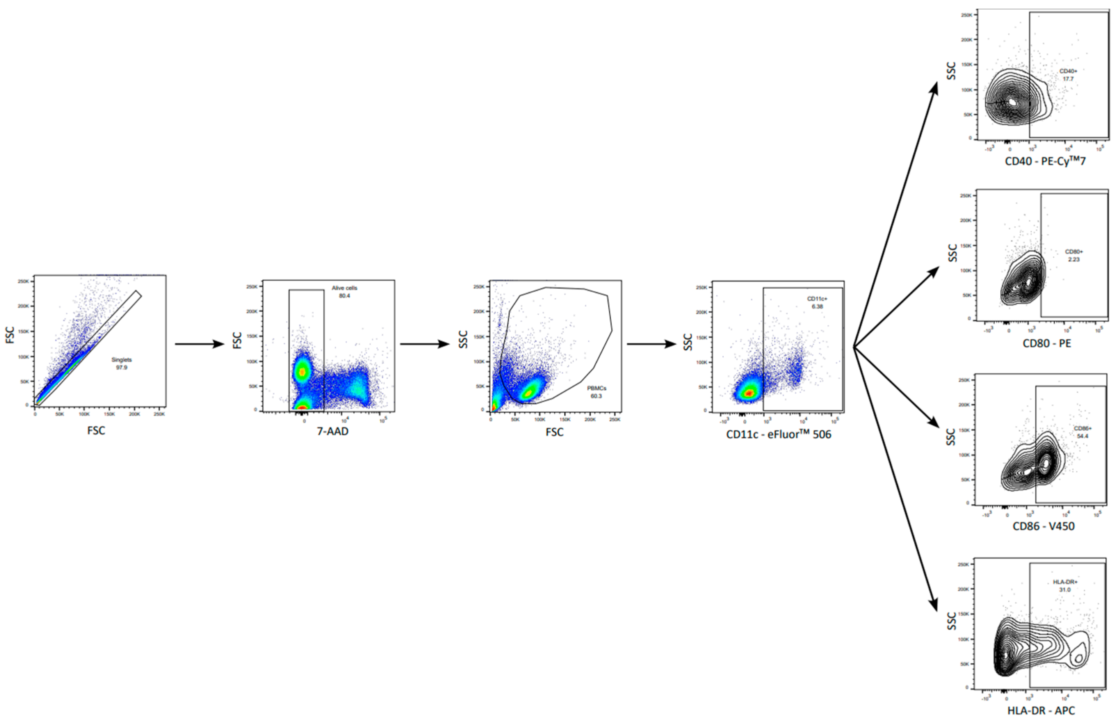

Figure S2 Gating strategy for determination of activation markers of CD11c+ cells from PBMCs after LNPs stimulation.
